# *ConversationAlign*: Open-source software for analyzing patterns of lexical use and alignment in conversation transcripts

**DOI:** 10.3758/s13428-026-02954-w

**Published:** 2026-02-20

**Authors:** Benjamin Sacks, Virginia Ulichney, Anna Duncan, Chelsea Helion, Sarah M. Weinstein, Tania Giovannetti, Gus Cooney, Jamie Reilly

**Affiliations:** 1 Department of Psychology and Neuroscience, Temple University, 1701 N. 13th Street, Philadelphia, PA 19122 USA; 2 Department of Communication Sciences and Disorders, Temple University, Philadelphia, PA USA; 3 Department of Epidemiology and Biostatistics, Temple University, Philadelphia, PA USA; 4 Department of Psychological and Brain Sciences, Dartmouth College, Hanover, NH USA

**Keywords:** Conversation analysis, Communication, Alignment, Language

## Abstract

Much of our scientific understanding of language processing has been informed by controlled experiments divorced from the real-world demands of naturalistic communication. Conversation requires synchronization of rate, amplitude, lexical complexity, affective coloring, shared reference, and countless other verbal and nonverbal dimensions. Conversation is not merely a vector for information transfer but also serves as a mechanism for establishing or maintaining social relationships. This process of language calibration between interlocutors is known as *linguistic alignment*. We developed an open-source R package, *ConversationAlign*, capable of computing novel indices of linguistic alignment and main effects of language use between interlocutors by evaluating word choice across numerous semantic, affective, and lexical dimensions (e.g., valence, concreteness, frequency, word length). We describe the operations of *ConversationAlign,* including its primary functions of cleaning and transforming raw language data into simultaneous time series objects aggregated by interlocutor, turn, and conversation. We then outline mathematical operations involved in computing complementary indices of linguistic alignment that capture both local (synchrony in turn-by-turn scores) and global relations (overall proximity) between interlocutors. We present a use case of *ConversationAlign* applied to interview transcripts from American radio legend Terry Gross and her many guests spanning 15 years. We identify caveats for use and potential sources of bias (e.g., polysemy, missing data, robustness to brief language samples) and close with a discussion of potential applications to other populations. *ConversationAlign* (v 0.4.0) is freely available for download and use via CRAN or GitHub. For technical instructions and download, visit https://github.com/Reilly-ConceptsCognitionLab/ConversationAlign.

## Introduction

In an ideal conversation, people modify the form and content of their own production to accommodate the unique demands and capacities of their interlocutor(s). This synchronization process, formally known as alignment, spans countless domains. For example, linguistic alignment involves selecting words and syntactic constructions tailored to the language proficiency of interlocutors, flexibly adjusting for perceived state-level factors (e.g., partner’s mood and perceived interest level) and trait-level individual differences (e.g., partner’s age and perceived social status; Fisher & Ram, 2024; Pickering & Garrod, 2006, 2021). Although it might be tempting to view conversation as a purely linguistic act, this perspective yields an incomplete picture of alignment as a multi-modal, social phenomenon (Hagoort & Özyürek, 2024) that can also be observed as changes in physical (e.g., gestures, facial expressions), neural, and linguistic patterns (Bergmann & Kopp, 2012; Louwerse et al., 2012; Sievers et al., 2024).

These patterns are all modulated to synchronize with the demands, capacities, and social norms of one’s interlocutor or audience. For example, expectations of formality (e.g., courtrooms) and reverence (e.g., churches) impose rules for linguistic alignment that govern what we say, how we say it, and who we say it to.

When successful, these adjustments lead interlocutors to better accommodate one another in various aspects of linguistic production through fluid changes and repairs over the course of the conversation (Giles et al., 1991; Ireland et al., 2011; Pickering & Garrod, 2004, 2021; Reitter & Moore, 2014). These active adjustments often pass back and forth between participants, gradually creating a shared understanding and facilitating the effective transfer of information between parties (Clark & Brennan, 1991). Linguistic alignment itself consists of several processes where interlocutors adjust syntactic, phonological, and lexical properties of their production (Ostrand & Chodroff, 2021). For example, if Mary tells John her “dog slept soundly on a cot last night”, John may align lexically (using the word cot instead of bed), syntactically (including two objects and an adverb in his sentence), or phonologically (shifting his own pronunciation of cot).

One perennial challenge of discussing alignment involves term specificity. The study of conversation has historically been undertaken by numerous subdisciplines (e.g., social psychology, communication, rhetoric, linguistics). Each of these fields has developed its own esoteric lexicon for referring to related phenomena. For example, when a linguist encounters the term 'word alignment,' they might assume the author is referring to syntactic alignment, a specific subset of linguistic alignment. In contrast, a communication theorist might link alignment with shared reference and common ground between interlocutors. Our reference to linguistic alignment here aligns with that proposed by Pickering and Garrod (2021) involving the tendency ofpeople to synchronize the lexical, semantic, morphological, and affective complexity of their language output (Srivastava et al., 2025).^1^

Most measurements of linguistic alignment can be described as either conditional (i.e., examining the change in word use between interlocutors at each turn) or distributional (i.e., comparing word use between interlocutors during the conversation as a whole; Doyle & Frank, 2016). Conditional metrics allow researchers to infer leader-follower dynamics in alignment (i.e., who aligns to whom). For example, if Mary utters an unpleasant utterance that is reciprocated by John, it can be concluded from this single exchange that John is aligning with Mary. Conditional metrics also discriminate between active linguistic alignment (e.g., Mary discusses something unpleasant, leading John to discuss unpleasant topics during the conversation) and baseline similarity in language use (e.g., John and Mary both discuss unpleasant things to a similar degree prior to the conversation; Doyle & Frank, 2016).

Measurement of linguistic alignment (hereafter abbreviated to alignment) has recently benefited from advances in deep learning and natural language processing. Embedding algorithms (e.g., BERT, ELMo) have been used to model syntax and semantics using probabilistic measures (Devlin et al., 2019; Peters et al., 2018). Each of these methods has strengths in modeling discourse properties for blocks of continuous text, such as those observed in narrative storytelling. Although embedding-based approaches and large language models are now being integrated into conversation analysis, the complexities of conversation (e.g., turn-taking, backchanneling, monopolizing, rapid topic shifting) pose challenges for computing unbiased measures of alignment (Rosen & Dale, 2024; Ward & Litman, 2007). Recently, programs have successfully applied natural language processing techniques to measure alignment at a turn-by-turn level (e.g., ALIGN library for Python; Duran et al., 2019), leveraging part-of-speech tagging and word embeddings to model turns with greater detail. *ConversationAlign* is a freely available R package that integrates the merits of past techniques within a flexible and semi-automated application capable of transforming vast amounts of dyadic conversation data into numeric time series on a scale that would ordinarily be prohibitive for manual coding systems (for a recent example of such a large-scale analysis, see Reilly et al., 2025).

*ConversationAlign* is not a large language model, nor does it rely on any proprietary generative artificial intelligence dependencies. The program instead capitalizes on the vast amount of publicly available word norms (e.g., age of acquisition, word length, frequency, valence) by tokenizing each target transcript and then prompting the user to select from among 45 possible dimensions for computing alignment and main effects. For example, when a researcher interested in toddler-directed speech is in dialogue with a child, a child with sufficient language competence will spontaneously align with the adult by producing more complex words-per-turn than their baseline language use. *ConversationAlign* is capable of quantifying alignment across numerous dimensions of lexical, semantic, phonological, and morphological complexity (e.g., morpheme count, word length, concreteness, word frequency) by yoking published norms to each tokenized word in the target transcript.

## Method

*ConversationAlign* (v 0.4.0) is an R software package capable of processing and analyzing patterns of language use and alignment in two-person (i.e., dyadic) conversation transcripts. In its current form, *ConversationAlign* is an English-only software application.^2^ The application is available for download and use either through CRAN or GitHub. For installation instructions and package documentation, visit https://github.com/Reilly-ConceptsCognitionLab/ConversationAlign.

*ConversationAlign* operates via a sequential processing pipeline where the output of each stage is piped into the next step. These operations ultimately transform each raw conversation transcript into a simultaneous time series aggregated by interlocutor and turn. The application first imports and formats one or more conversation transcripts into R, automatically assigning a unique identifier to each transcript and binding all individual transcripts into a single data frame. The next steps (i.e., cleaning and formatting) yield an ordered vector of words to which the software appends word norms for up to three choices from a total of 45 affective, phonological, lexical, and semantic dimensions (Brysbaert et al., 2014, 2024; Brysbaert & New, 2009; Gao et al., 2023; Hoffman et al., 2013; Keuleers et al., 2015; Kuperman et al., 2012; Lynott et al., 2020; Martínez et al., 2024; Miller, 1995; Mohammad, 2018; Raji & de Melo, 2020; Sánchez-Gutiérrez et al., 2018; Shaoul & Westbury, 2010). *ConversationAlign* indexes these norms to compute main effects and alignment indices between partners within each conversation.

## Preparing for your conversation analysis

*ConversationAlign* was designed to be accessible to researchers without extensive expertise in computer science or computational linguistics. Although the software contains many fail-safes and error checks, users should be aware of its potential pitfalls. Careful planning and hypothesis-driven contrast selection are essential. A well-articulated file management strategy (e.g., naming conventions, version control) is critical for managing the proliferation of data that *ConversationAlign* will inevitably yield with each new processing step. Each conversation transcript must be saved as a separate text (.txt) or comma-separated value (.csv) file with a unique filename. Users should be deliberate in their file naming conventions, as *ConversationAlign* appends each transcript’s filename as an identifier (i.e., ‘event_id’) within a data frame organizing every conversation.

*ConversationAlign* scans a designated folder on the user’s computer for all raw (i.e., unprocessed) conversation transcripts. During the read step, users are prompted to specify a path if using a custom folder name (e.g., ‘../MyBigProject/Conversation_Transcripts’). Each conversation transcript must nominally contain at least two columns: 1) ‘participant_id’: a participant identifier that specifies who is producing each utterance; 2) text: the corresponding transcribed utterance. *ConversationAlign* preserves metadata such as timestamps and concurrent physiological measurements (e.g., pupil diameter, head motion parameters, gaze coordinates) that are commonly used in hyperscanning and other two-person neuroscience applications.

## Import your transcripts into R using 'read_dyads()'

The first processing step undertaken by *ConversationAlign* involves importing and formatting user-specified data with a custom function `read_dyads()`. This function scans a designated directory path (i.e., folder) supplied by the user, extracts all files (.csv or .txt) in the folder as a list into the R workspace, checks for non-standard character encoding (non-UTF-8), and concatenates all conversation transcripts into a single data frame. A key part of the step involves extracting the individual names of each .txt or .csv file and appending these filenames as unique event identifiers that mark each conversation as distinct levels of a factor variable (for grouping). `read_dyads()` will retain any associated metadata. Turns and exchanges are automatically marked within each conversation using `consecutive_id()` from *dplyr* that increments with every switch of the level of the ‘participant_id’ (see Section 2.3; Wickham et al., 2023).

## Clean and format transcripts using 'prep_dyads()'

After importing a conversation corpus, users have many options for cleaning their data. The software splits a raw language transcript into a one-word-per-row format, appending norms to each token via a database join. Raw language transcripts are often noisy, containing many hidden characters, non-alphabetic symbols, and transcription errors. Users interested in isolating open-class (i.e., content) words must typically execute a series of cleaning steps. `prep_dyads()` applies several obligatory text-cleaning transformations, along with optional arguments such as stopword removal and lemmatization. All strings are first converted to lowercase, and non-standard apostrophes are replaced using the R *textclean* package (Rinker, 2018a). These standardization procedures are essential for term aggregation and matching corresponding strings in *ConversationAlign*’s lookup database. The software then loops over each row of the transcript, evaluating matches in a static list of contractions and executing replacements (e.g., don’t becomes do not, isn’t becomes is not)^3^. `prep_dyads()` contains additional arguments, including lemmatization (i.e., converting morphological derivatives of words to their dictionary forms; (Rinker, 2018b) and stopword removal.

Stopword removal is a nontrivial aspect of discourse analysis. Overly liberal removal criteria can potentially bias interpretation by omitting open-class words. *ConversationAlign* (v 0.4.0) includes several different stopword lists that vary by ‘strictness’ of the removal criteria. The default stopword list for *ConversationAlign* omits determiners (e.g., *the, a*)*,* adpositions (e.g., *on, in*)*,* filler words (e.g*., uh, um*), pronouns (e.g*., he, she,* *it*), Arabic numerals (*0–12*), modal auxiliaries (e.g., *should*), idioms, and greetings (e.g., *hello, how’s it going*). Further details about stopword options, including the derivation of the default stopword list, can be found on the project’s OSF page, along with the default list (‘Temple_stop25’) itself. This list can be inspected at https://osf.io/bkzre/overview.

After text cleaning, stopword removal, and lemmatization, `prep_dyads()` prompts its user to enter at least one and at most three dimensions of interest for computing alignment in a given analysis. The program uses these selections to map lexical norms from each vectorized content word in the transcript to its corresponding norms in a custom lookup database using a `left_join()` (Wickham et al., 2023). This internal database (‘lookup_Jul25’) comprises many English tokens (*N* = 102,682) across 45 dimensions. We constructed this composite lookup database by extracting norms from other previously published psycholinguistic databases' dimensions (Brysbaert et al., 2014, 2024; Brysbaert & New, 2009; Gao et al., 2023; Hoffman et al., 2013; Keuleers et al., 2015; Kuperman et al., 2012; Lynott et al., 2020; Martínez et al., 2024; Miller, 1995; Mohammad, 2018; Raji & de Melo, 2020; Sánchez-Gutiérrez et al., 2018; Shaoul & Westbury, 2010).

A variable key for *ConversationAlign* (v 0.4.0) with descriptions, ranges, and sources of each dimension can be found as supplementary material in the project’s OSF repository at https://osf.io/bkzre/overview (or view an HTML version of the variable key directly at https://reilly-lab.github.io/ConversationAlign_LookupJul25_VariableKey) along with code detailing how we derived this lookup database.

*ConversationAlign* (v 0.4.0) automatically appends a sequential ‘turn count’ to each utterance by looping over the target data frame and counting all strings produced by one interlocutor as components of a single turn. When the level of ‘participant_id’ changes, denoting a speaker shift, *ConversationAlign* increments the turn count by one. Thus, the software delineates turns as all language produced by one interlocutor until there is a change in interlocutor. We operationally defined an exchange as a discrete back and forth over two successive turns. *ConversationAlign* reports missing values (e.g., no database matches on a given norm, turn consists entirely of omitted stopwords) as NAs.

*ConversationAlign* replaces stopwords with NAs row-wise, providing two options for handling missing data. When the argument ‘remove_backchanneling’ in `prep_dyads()` is set to true, *ConversationAlign* removes rows with words that have been dropped and replaced with NA during the cleaning process. If an entire turn consists of stopwords, as is often the case for backchanneling (e.g., “oh yeah”), the turn will be removed, and the preceding turn will be merged with the next non-backchannel turn. This procedure reduces the impact of backchanneling but will also inevitably shorten the transcript. Alternatively, if the user wishes to retain backchannel responses (i.e., remove_backchanneling = false), *ConversationAlign* will retain and interpolate over NAs prior to analysis (see section 2.5.1). We recommend that both individual transcripts and corpus analytics be examined prior to determining how NAs replacing stopwords are handled.

## Generate corpus analytics: 'describe_corpus()'

Discourse sampling is vulnerable to numerous sources of error and systematic bias (e.g., regional sampling limited to a single demographic population). Corpus analytics can therefore provide a critical window into representativeness. Not only is it important to quantify characteristics of who is talking, but also the nature of what is being said. Numerous R packages contain functions for computing relevant text analytics, such as type-token ratio and word counts. *ConversationAlign* (v 0.4.0) includes a built-in function `describe_corpus()` specifically tailored to the unique nature of dyadic conversation transcripts. `describe_corpus()` automatically generates a near publication-ready table of analytics spanning raw and cleaned samples. The table generated by `describe_corpus()` includes descriptive statistics (e.g., mean, range, standard deviation) for turn count, word count, cleaning retention rate, morphemes-per-word, lexical frequency, words per turn, and type-token ratio.

## Computing alignment indices and main effects: `summarize_dyads()`

The final stage of *ConversationAlign* quantifies alignment between interlocutors using the time series structure (i.e., by exchange) with the function `summarize_dyads()`. When called, `summarize_dyads()` computes two complementary indices of alignment. Area under the difference curve (dAUC) reflects a measure of proximity (e.g., at any given time point, how similar are John’s production and Mary’s production?). In contrast, interlocutor synchrony (e.g., how immediately responsive is Mary when John utters something unpleasant?) is measured using correlations. Correlations may also be led or lagged to examine leader–follower dynamics (e.g., does John adjust more to Mary than Mary adjusts to John?; for further discussion on various descriptors of alignment, see daSilva & Wood, 2025). While each metric uses individual turn values in its calculations, it reports a single value that describes alignment over the course of the entire conversation (i.e., one value per conversation). *ConversationAlign* generates alignment indices for each specified dimension of interest.

Users should exercise caution when analyzing brief conversations (< 50 turns) or conversations where one interlocutor monopolizes while their partner backchannels (e.g., “um hm… really?”; see also Bonett & Wright, 2000; David, 1938). *ConversationAlign* treats most backchannel responses as stopwords. *ConversationAlign* warns users when any conversation transcript contains fewer than 50 turns.

## dAUC (area under the difference curve)

**Fig. 1:**
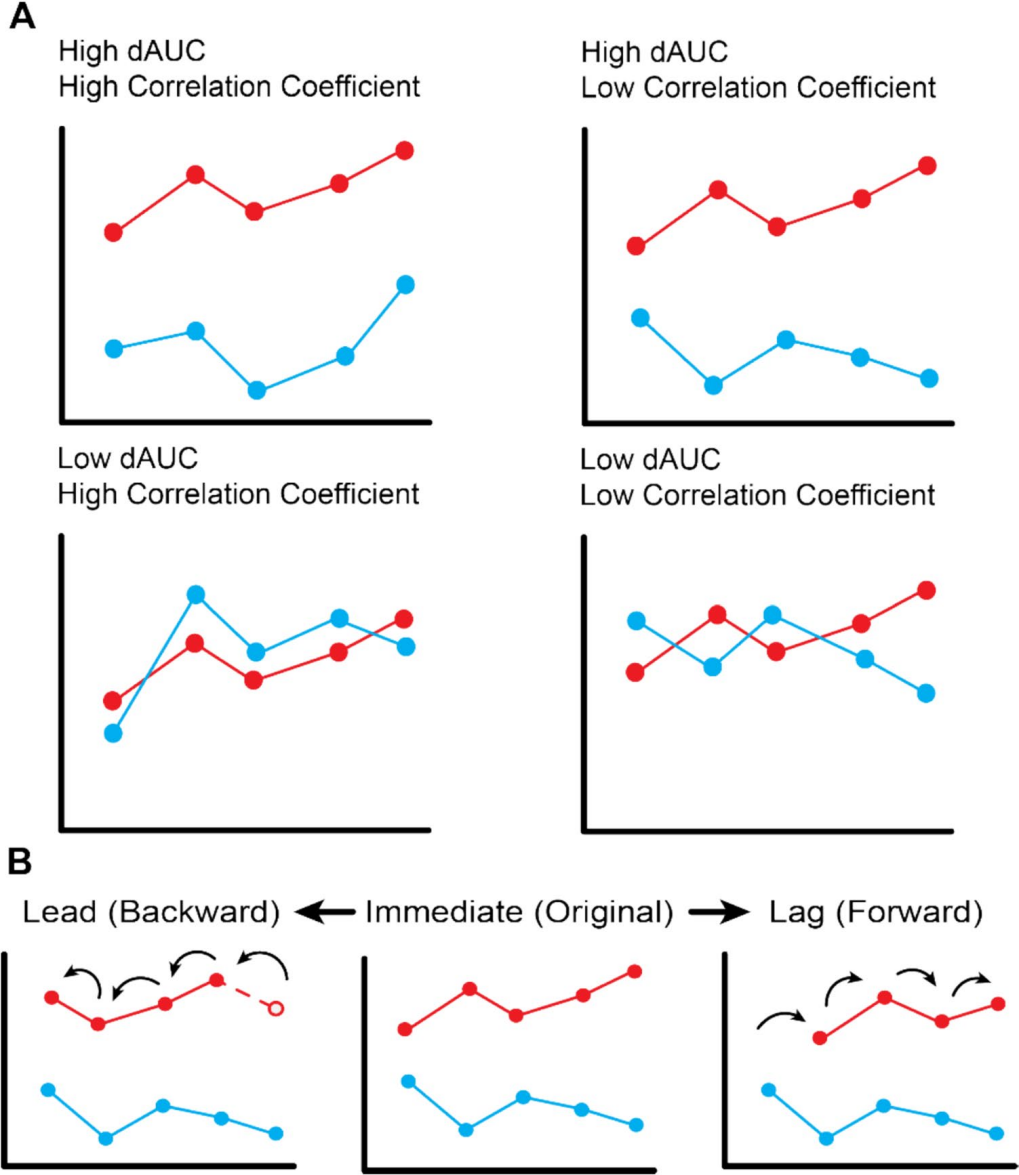
Example time series associated with varying magnitudes of dAUC and covariance computed by *ConversationAlign. Note*: Area under the difference curve (dAUC) and correlation coefficients provide two distinct measures of alignment. A higher magnitude dAUC rating represents greater distance (lower proximity) between interlocutor scores, but does not provide information on synchrony between interlocutors. Meanwhile, a high magnitude correlation coefficient represents high interlocutor covariance, or similar changes in production at the same turns, but does not provide information on the proximity of interlocutor scores at each point. Taken together, these metrics provide several unique scenarios for alignment within each dyad (**A**). An interlocutor’s time series may be offset to examine if one interlocutor is ‘leading’ and another is ‘following’ trends in production. For each supplied lead or lag, a single interlocutor’s time series is shifted forward or backward. Within each conversation, the first interlocutor to speak is offset (**B**)

Area under the difference curve (dAUC) represents a measure of proximity (i.e., distance in turn ratings) between two conversation partners on any target dimension over the course of a conversation. Proximity versus directionality in alignment can be illustrated in a conversation between two people (Mary and John) who are both expressing anger in lockstep. However, John and Mary differ in the intensity of their expression. Mary tends to curse, whereas John’s expressions of anger are more muted. In this case, Mary and John might show high turn-by-turn synchrony (i.e., adjust in a similar direction to one another) in that both are expressing anger. However, the distance between Mary and John at any given turn is likely higher. These two indices, therefore, quantify different aspects of alignment (Fig. 1).

When two partners are close on a given dimension (e.g., valence), the area between them will be relatively low. *ConversationAlign* computes the absolute value of the difference between interlocutors at each turn, collapsed across all words. This difference time series must be continuous in order to derive a dAUC value. Missing observations from either interlocutor’s time series (i.e., a turn without values on a certain dimension, denoted as ‘NA’) must be filled or interpolated prior to calculating the difference time series. Prior to generating the difference time series, missing observations at the beginning or end of the interlocutor’s time series are filled with the closest reported value using *tidyr* (Wickham et al., 2024), while missing values in the middle of the series are filled with linear interpolation using the R package *zoo* (Zeileis & Grothendieck, 2005). The resulting continuous time series are used to compute the absolute difference time series, which is provided as input to calculate dAUC with the function `AUC()` from *DescTools*, using the trapezoidal rule (Signorell et al., 2024).

dAUC is a relative measure whose magnitude is unique to each conversation and dimension. The values of dAUC as derived by *ConversationAlign* grow in tandem with turn length, even if the global difference between two conversation partners remains constant. This phenomenon is best illustrated by another hypothetical conversation between John and Mary. This conversation contains just one repetitive turn where John says the word *happy*, and Mary responds with *sad*. The distance between *happy* and *sad* on any given turn is a constant. If one wished to measure proximity between John and Mary on happiness, they would hope to capture this constant difference. However, raw dAUC is also influenced by conversation length, as one parameter of the area calculation is ‘turn_count’. *ConversationAlign* (v 0.4.0) outputs two dAUC values for each target dimension, a raw dAUC and standardized dAUC. Raw dAUC is computed by the trapezoidal rule, summating absolute differences across all turns regardless of the length of the conversation. Normalized dAUC is derived by converting the raw dAUC to a standardized turn length (50 turns) as shown by the following equation:

$$\frac{dAUC\;\left(observed\right)}{turn\;count\;\left(observed\right)}=\frac{dAUC\;normalized}{50\;turns}.$$

In addition to conversation length, dAUC is also sensitive to ordering of turns. In original turn pairings, the first interlocutor to speak will always precede the second interlocutor, leading the dAUC to disproportionately weight the second interlocutor’s alignment to their partner. To account for this, *ConversationAlign* reports dAUC values calculated from time series pairings lagged by one turn, switching the order of interlocutor turns within each exchange (i.e., the second interlocutor to speak originally now precedes the first). The interlocutor who originally spoke first is reported to track directionality. dAUC calculations are joined to the original data frame by document identifier and reported between interlocutors for each conversation and psycholinguistic dimension.

## Spearman and Pearson correlations

*ConversationAlign* quantifies the strength of synchrony (i.e., turn-by-turn covariance) between interlocutors using lagged Spearman or Pearson correlations. Synchrony between interlocutors can be illustrated by returning to the same hypothetical conversation between John and Mary. If John always responds pleasantly to a pleasant utterance from Mary, it can be said that turn-by-turn alignment on valence between John and Mary is strongly positively correlated. *ConversationAlign* computes indices of turn-by-turn alignment by first interpolating over each interlocutor’s time series (see 2.5.1). The program then derives Spearman or Pearson correlations at several default lags, with additional lags specified as an argument to the function call.

Pearson correlations require data to be normally distributed, an assumption that is not always met in language data. The Spearman correlation for each conversation provides a nonparametric (ordinal-rank) measure of synchrony between two interlocutors across all exchanges in a conversation for each dimension of interest. Users may select the method used for computing correlations using the argument ‘corr_type’ when calling `summarize_dyads()`. One possibility is that linguistic alignment does not occur immediately (e.g., John is pleasant in response to Mary) but after some lag (e.g., John is pleasant in response to Mary but only after an intervening delay of three turns). To account for this, *ConversationAlign* measures this type of turn-by-turn variance via lagged correlations.

To compute lagged correlations, *ConversationAlign* shifts the time series of a single interlocutor. The first interlocutor to speak in each conversation is shifted, with their identifier reported in an additional column for reference. If negative lags are supplied, they are treated as leads, and the time series is shifted ahead. Time series are lagged, and correlations are computed using the function `cor.lag()` from *YRMisc* (Russon & Yin, 2020). For Spearman correlations, scores are converted to ranks immediately preceding correlation calculations with the same function. Both Spearman and Pearson correlation coefficients range from negative one to positive one, describing the magnitude and direction of the association (Lee Rodgers & Nicewander, 1988; Zar, 2005).

Similar to dAUC, correlations rely on turn pairings and will also disproportionately weight the second interlocutor to speak in each pair. If John speaks first, then in each pairing, Mary’s response to John is paired with John’s statement, but John’s response to Mary is grouped into the following turn. This pairing fails to consider John’s responses to Mary. A lag of one flips the order of interlocutors within each exchange, now grouping John’s responses to Mary with her original statements. These values may be compared to assess interlocutor differences in alignment, or averaged for a holistic measure of synchrony in alignment for the whole conversation. The correlation at each specific lead and lag is reported in a separate column, organized by conversation and psycholinguistic dimension. dAUC and correlations assess two unique aspects of alignment, shown in Fig. 1.

## Creation of sham control time series

To complement the analyses included in `summarize_dyads()`, *ConversationAlign* allows users to generate permutations of each individual dyad. When `generate_shams()` is called, the order of both interlocutors’ turns within each dyad is shuffled. This removes the continuity of the conversation (i.e., turn 2 no longer inherently follows turn 1). `generate_shams()` accepts the output data frame from `prep_dyads()` and offers the option for users to provide a seed for reproducibility. The resulting data frame with sham controls is formatted and prepared for input into `summarize_dyads()`. Metrics of alignment (dAUC and correlations) computed from shuffled dyads provide “ground truth” information about the strength of alignment without the influence turn-to-turn adjustments between interlocutors. Shuffled dyads will contain information about global alignment (i.e., how interlocutors influence each other over the course of the whole conversation). Thus, sham dyads act as a control for a variety of research questions and allow for the separation of turn-to-turn alignment effects from global alignment or baseline scores.

## Analysis: example use case

### Development of Terry Gross interview corpus

We compiled a corpus of interviews (*N* = 60) between longtime host Terry Gross and various guests from National Public Radio’s show *Fresh Air* (*Fresh Air* *Archive*, 2001). Each interview consists of naturalistic dialogue between Terry Gross and a single guest. We accessed transcripts from the *Fresh Air* Archive and saved each interview transcript as a .txt file for further processing (*Fresh Air* *Archive*, 2001). We randomly selected four transcripts from the collection of recordings for each year between 2010 and 2024, excluding interviews hosted by interviewers other than Terry Gross (e.g., Tonya Mosley) and interviews featuring more than one guest. From each year, we collected interviews from two guests who use she/her pronouns and two guests who use he/him pronouns. Transcripts with fewer than 30 exchanges after cleaning and aligning with *ConversationAlign* were excluded to ensure the accuracy of dAUC and Spearman correlation results. *Fresh Air* interviews are long-form, with extended turns and sparse backchanneling compared to a standard conversation. Given this information, we consider the 30-exchange cutoff appropriate for the 50-turn standardization of dAUC values as well. The recording year and pronouns used by the guest for each of the 60 transcripts were stored in a separate metadata file.

Prior to reading transcripts with *ConversationAlign*, we imported each .txt file into R to clean and format each transcript into a tabular format. This was accomplished by reading each transcript line-by-line and splitting the interlocutor and utterance, delimited by a colon, into separate columns of a data frame. Utterances delimited into multiple lines were collapsed into a single string to better group utterances for exclusion. We then used a series of regular expressions for additional pre-processing—specifically, to remove any whole utterances containing language data interrupting the conversation. Markers of these interruptions include “(As”, demonstrating actor portrayals, as well as “(Singing)” and “(Reading)”, denoting a live performance from the guest. Next, any text within parentheses or quotations was removed, splicing out laughter, sound clips, and titles. Additionally, each interview contains several breaks for commercials, preceded and followed by an introduction by Terry Gross. These interruptions were excluded from the transcript by removing utterances containing the string “FRESH AIR” or “my guest is”. Finally, we removed all non-alphanumeric and non-punctuation characters and removed strings comprised only of whitespace and punctuation left over from formatting. To confirm that each interlocutor was referred to by a single title, we replaced the full name of either interlocutor with the more commonly used last name. The resulting formatted and cleaned transcripts were written as .csv files with two columns: Interlocutor and Text. Transcripts from the *Fresh Air Archive* (*Fresh Air Archive*, 2001), metadata, and code for cleaning are available for view or download at https://osf.io/bkzre/.

## Analysis

We investigated changes in lexical production and linguistic alignment between Terry Gross and her many interview guests over a 15-year period (2010 to 2024). We specifically evaluated whether linguistic valence and semantic concreteness^4^ systematically differed between Gross and her guests and whether these differences also influenced alignment (Martínez et al., 2024). We used *ConversationAlign*’s standard pipeline for processing these dyadic transcripts (details in Table 1). We determined that stopwords should be preserved as NAs due to the structure of the interview format, with long turns and minimal backchanneling. The average number of turns per conversation consisting entirely of NAs after cleaning was 1.66. The low frequency of fully NA turns (i.e., turns requiring interpolation) within the corpus leaves little room for interpolation to alter results away from real observations.

**Table 1:** Description of the Terry Gross interview corpus

Measure	Mean	Stdev	Min	Max
Total number of conversations	60.00			
Token count all conversations (raw)	349,271			
Token count all conversations (post-cleaning)	174,057			
Exchange count (by conversation)	46.08	15.13	31.00	97.00
Word count raw (by conversation)	5821.18	938.86	4,221.00	7973.00
Word count clean (by conversation)	2900.95	478.07	2177.00	4122.00
Cleaning retention rate (by conversation)	0.50	0.02	0.46	0.55
Morphemes-per-word (by conversation)	1.20	0.05	1.12	1.35
Letters-per-word (by conversation)	4.89	0.22	4.54	5.56
Lexical frequency lg10 (by conversation)	4.18	0.15	3.82	4.42
Words-per-turn raw (by conversation)	68.94	20.08	26.32	112.60
Words-per-turn clean (by conversation)	34.44	10.42	12.95	58.89
Type-token ratio raw (by conversation)	0.18	0.02	0.15	0.24
Type-token ratio clean (by conversation)	0.27	0.03	0.22	0.36

Table of descriptive statistics detailing the length and preservation of words after cleaning within the Terry Gross interview corpus. This table was generated using ‘corpus_analytics()’ included in *ConversationAlign*. Cleaning retention rate describes the proportion of words present after cleaning each transcript.

We then used *ConversationAlign* to compute dAUC and correlations with time series offsets (i.e., leads or lags) ranging from three turns ahead to three turns behind. We averaged both standardized dAUC values from each conversation (immediate and lagged one turn) to generate a single value that considers both ways of pairing interlocutor responses. All comparisons were performed on both ratings of concreteness and linguistic valence. We generated sham conversations using ‘generate_shams()’ and computed dAUC with the same method. We first compared dAUC values from real conversations to shams using a Wilcoxon rank-sum test. Effect size (r) was calculated from the standardized test statistic (*z*) divided by the square root of the number of pairs (i.e., interviews). We used a second Wilcoxon rank-sum test to assess the difference in dAUC between dyads based on the pronoun set used by the guest.

Next, we compared the immediate correlations (i.e., no lag or lead) of real and sham conversations using a Wilcoxon rank-sum test. Within each conversation, the first interlocutor to speak is selected as the time series to offset. To compare correlations at various offsets, we first had to standardize each conversation as if Terry Gross were the first to speak. We accomplished this by switching lag and lead values in conversations where Terry Gross was the second interlocutor to speak. We then compared correlations from various offsets with a Kruskal-Wallis test and post-hoc Dunn’s test with Bonferroni correction for individual comparisons (Dunn, 1964). We used the package *dunn.test* to perform Dunn’s test (Dinno, 2024). Finally, we performed three Wilcoxon rank-sum tests to compare interlocutor synchrony (turn-by-turn covariance) between conversations by the guest’s used set of pronouns with Terry Gross led one turn ahead, lagged one turn, and with no offset.

## Results

### Area under the difference curve (dAUC) did not differ significantly between sham and real conversations, nor between conversations with guests using she/her pronouns and guests using he/him pronouns

**Fig. 2:**
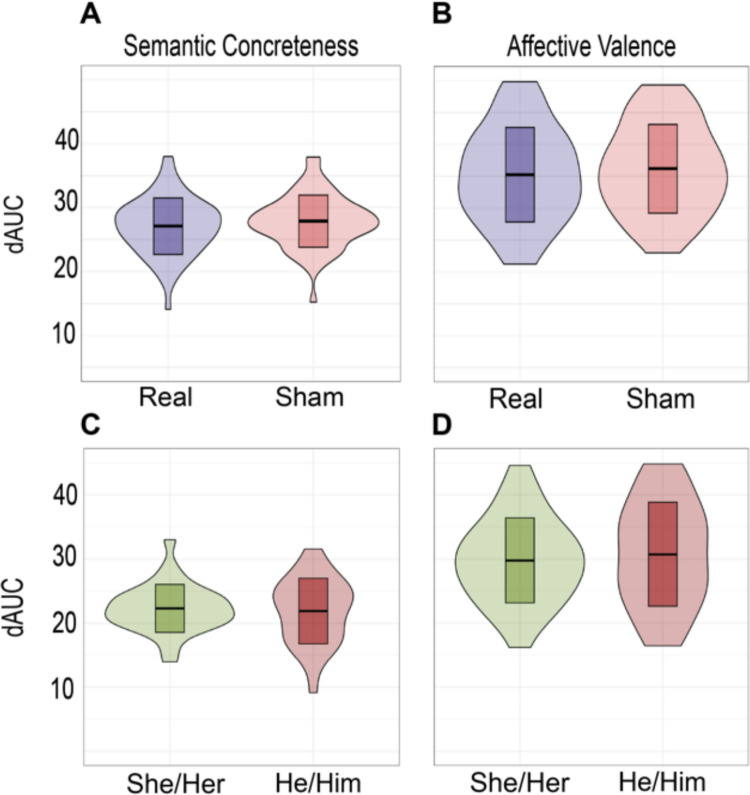
Group differences in alignment measured in area under the difference curve (dAUC). Area under the difference curve (dAUC) did not differ between real and sham conversations for ratings of semantic concreteness (**A**) or affective valence (**B**). Similarly, dAUC did not differ between conversations based on the pronouns used by the guests for semantic concreteness (**C**) or affective valence (**D**). *Center bars* represent the mean. Shown with *error bars* representing one standard deviation surrounding the mean

We found that dAUC values did not differ significantly between real and sham conversations on ratings of concreteness, *z* = – 0.890, *r* = 0.115, *p* = 0.374 (Fig. 2A) or valence, *z* = – 0.664, *r* = 0.0857, *p* = 0.507 (Fig. 2B). Similarly, we found no significant difference in dAUC between conversations with guests using she/her pronouns or guests using he/him pronouns when comparing concreteness, *z* = 0, *r* = 0, *p* = 1 (Fig. 2C) or valence, *z* = – 0.492, *r* = 0.0899, *p* = 0.623 (Fig. 2D).

### Synchrony between ratings of linguistic valence is greater in real conversations compared to sham conversations

**Fig. 3:**
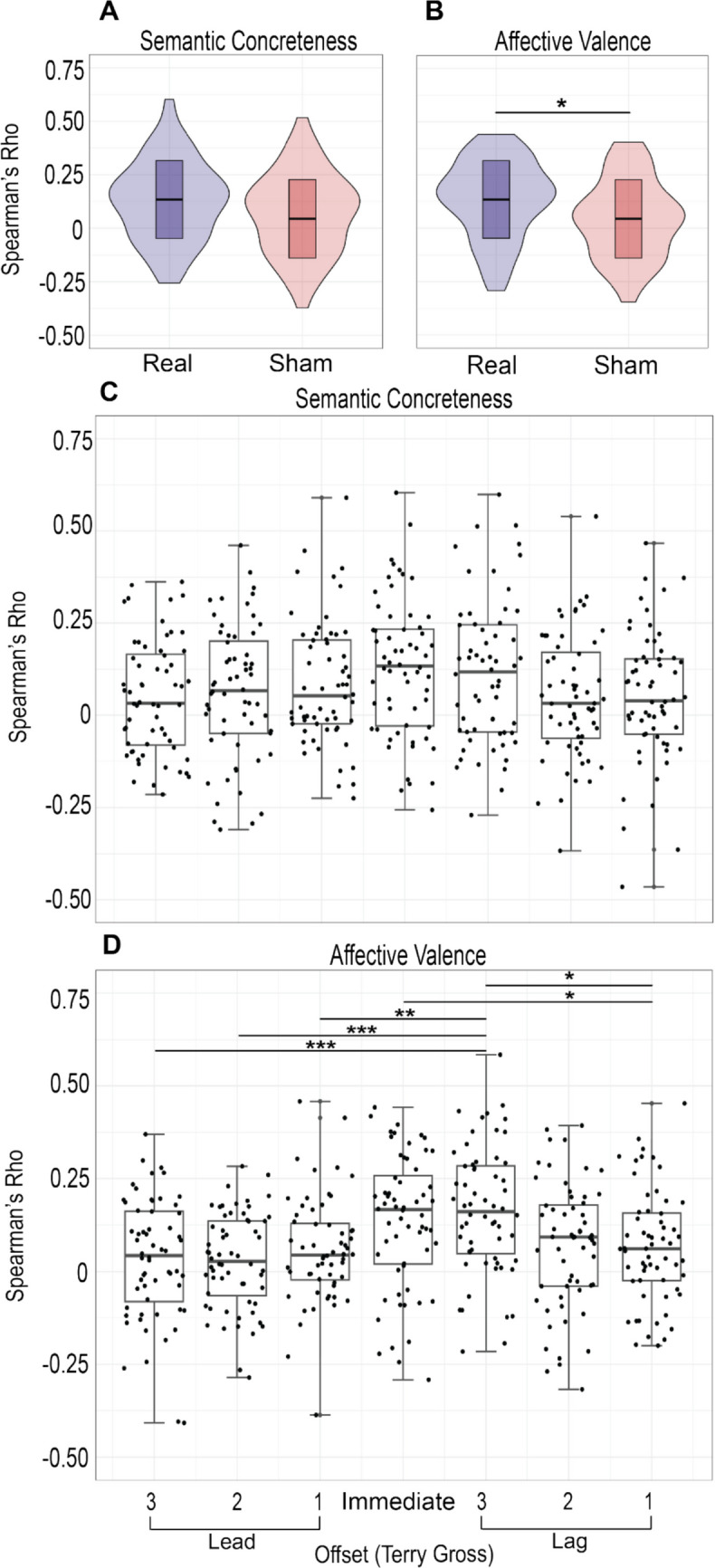
Comparison of interlocutor rating alignment at several offsets. No significant difference was observed in synchrony between interlocutors on ratings of semantic concreteness between real and randomly shuffled conversations (**A**). However, real conversations, on average, showed greater interlocutor synchrony on ratings of affective valence compared to randomly shuffled conversations (**B**). Spearman correlations were compared between offsets (i.e., leads or lags) shifting Terry Gross’s time series from a range of three turns ahead to three turns behind. Spearman correlation coefficients for ratings of semantic concreteness showed no significant differences between offsets (**C**). For ratings of affective valence, correlation coefficients were, on average, greater at a lag of one turn compared to a lead one turn, two turns, and three turns. Synchrony between interlocutors at an offset of one turn behind or immediate was also greater than at a lag of three turns behind (**D**). * = *p* < 0.05, ** = *p* < 0.01, *** = *p* < 0.001

When testing synchrony between Terry Gross and interview guests, we first generated sham conversations. We found no significant difference in the immediate Spearman correlations between concreteness scores from real and sham conversations, *z* = – 1.68, *r* = 0.217, *p* = 0.093 (Fig. 3A). However, we observed that real conversations showed larger immediate Spearman correlations for affective valence, on average, compared to sham conversations, *z* = – 2.82, *r* = 0.36, *p* = 0.0047 (Fig. 3B).

### Synchrony of interlocutor linguistic valence scores is greater when Terry Gross’s responses are lagged compared to guests

To assess leader–follower dynamics in alignment between Terry Gross and interview guests, we examined interlocutor synchrony at several leads and lags. We found no difference in synchrony for scores of concreteness across seven offsets, χ^2^(6, *N* = 7) = 10.35, *p* = 0.111 (Fig. 3C). However, we observed a difference in interlocutor synchrony for scores of valence across the same seven groups, χ^2^(6, *N* = 7) = 33.66, *p* < 0.001 (Fig. 3D). A subsequent Dunn test with Bonferroni correction revealed that synchrony between Terry Gross and guests was significantly higher when Terry Gross was lagged one turn compared to led one turn, *z* = – 3.54, *p* = 0.004, two turns, *z* = – 4.38, *p* < 0.001, and three turns ahead, *z* = – 3.96, *p* < 0.001. Synchrony between guests and Terry Gross was also significantly higher when Terry Gross was lagged one turn compared to lagged three turns, *z* = 3.24, *p* = 0.013, or when immediate correlations were compared to those lagged three turns, *z* = – 3.29, *p* = 0.010, showing a general trend of synchrony decreasing at larger offsets (Fig. 3D).

### Synchrony between Terry Gross and guests on scores of semantic concreteness is higher at a lag of one turn for conversations with guests using she/her pronouns and guests using he/him pronouns

Due to the decline in interlocutor synchrony observed at leads and lags of two or greater, we determined that correlations between pairing of immediate turns, Terry Gross lagged one turn, and Terry Gross led one turn should be compared. Synchrony between Terry Gross and guests on scores of concreteness was not significantly different at immediate turn pairings, *z* = – 3.82, *r* = 0.0282, *p* = 0.877 or pairings with Terry Gross led one turn, *z* = – 0.154, *r* = 0.0282, *p* = 0.877. Interlocutor synchrony on scores of concreteness did not differ significantly between conversations with guests using she/her pronouns and guests using he/him pronouns when Terry Gross was lagged one turn behind, *z* = – 2.01, *r* = 0.368, *p* = 0.0441. For synchrony between interlocutors on scores of valence, no significant differences were observed for immediate turn pairings z = – -1.02, *r* = 0.187, *p* = 0.306, pairing with Terry Gross led one turn, *z* = – 0.670, *r* = 0.122, *p* = 0.503, and pairing with Terry Gross lagged one turn, *z* = – 1.04, *r* = 0.190, *p* = 0.299.

## General discussion

We introduced *ConversationAlign* (v 0.4.0), a novel, open-source workflow for transforming dyadic transcripts into time series data showing average usage of psycholinguistic dimensions by turn and interlocutor in conversations. Included in this package are multiple methods for quantifying linguistic alignment (e.g., difference area under the curve or dAUC and lagged Spearman or Pearson correlations). To demonstrate these methods, we tested differences in alignment on ratings of linguistic valence (i.e., pleasantness) and semantic concreteness (Martínez et al., 2024) between real and sham conversations, as well as by the pronouns used by the interview guest.

From these analyses, we found that scores for dAUC did not differ between real and sham conversations. This may be due to a lack of large shifts in topic across the conversation that prevent the shuffling of conversations to drastically alter any two compared turns. We also found that dAUC did not differ between conversations with guests using she/her pronouns or guests using he/him pronouns. However, synchrony between interlocutors on ratings of linguistic valence was greater for real conversations compared to shams. Additionally, we found that interlocutor synchrony on ratings of valence was significantly higher when Terry Gross’s turns were lagged by a single turn relative to being led one, two, or three turns ahead. A lag of one emphasizes Terry Gross’s responses to her interview guests, and the higher synchrony suggests that Terry Gross altered her own word choice to match her guests to a greater extent than the converse. Additionally, we found that interlocutor synchrony at a lag of one turn on ratings of concreteness was greater in conversations with guests using she/her pronouns compared to guests using he/him pronouns.

These results demonstrate various approaches for assessing linguistic alignment with *ConversationAlign*. As shown, the included summary statistics provide accessible metrics of linguistic alignment over the course of an entire conversation. These values may serve as outcomes for an independent variable (e.g., guest pronouns) or as predictors (e.g., to test whether alignment on psycholinguistic dimensions is associated with social, cognitive, emotional predictors) in subsequent analyses. Additionally, unaggregated time series data generated after matching psycholinguistic values from databases to transcripts provide the foundation to take a more granular approach to measuring linguistic alignment through time series. By assessing linguistic alignment on these psycholinguistic dimensions, *ConversationAlign* provides a unique perspective and may supplement other measures that calculate such alignment using the frequency of specific words or word categories (Doyle & Frank, 2016). This builds on previous measures investigating dyadic interactions as time series and metrics of linguistic alignment (Doyle & Frank, 2016; Solomon et al., 2021; Xu & Reitter, 2015). Combined, *ConversationAlign* provides a novel algorithm for assessing linguistic alignment in dyadic conversations with both built-in metrics and options for further analysis.

## Limitations and caveats

*ConversationAlign* represents a potentially powerful tool for measuring the dynamics of alignment in conversation transcripts. However, users must also be aware of its limitations and potential sources of bias associated with its use. In reality, conversation is a multimodal phenomenon involving coordination of countless social, physical, and linguistic cues (e.g., physical proximity, body kinematics, prosodic variability, eye contact; Ostrand & Chodroff, 2021; Schoot et al., 2019; Bergmann & Kopp, 2012; Louwerse et al., 2012). *ConversationAlign* in its current form (v 0.4.0) computes indices of linguistic alignment by indexing properties of single words extracted from their original phrase and sentence contexts. Although the software is capable of flexibly integrating many other user-guided parameters, its lexical focus yields a sparse picture of a much more complex phenomenon. Some of the usage caveats for *ConversationAlign* are common to all methods of discourse analysis, such as the necessity for sampling large and diverse corpora and paying careful attention to inferencing and representativeness. Short conversation samples (< 50 turns) are particularly susceptible to bias when using our alignment algorithms, particularly in cases where one interlocutor consistently monopolizes turns while the other interlocutor backchannels (e.g., uh hm…), producing very few content words. Although we have recommended a minimum ‘safe’ threshold for deriving alignment measures (> 50 exchanges), small sample bias should be treated as an essential design consideration.

Another limitation of *ConversationAlign* (v 0.4.0) is that it is an English-only software platform. This focus on English was not an exclusionary design feature but a constraint imposed by the availability of lexical norms and validated text-cleaning tools (e.g., stopword lists, lemmatization functions) across other languages. We hope to expand *ConversationAlign*’s natural language scope as norms become more widely accessible. The pace of open-source lexical norm dissemination for languages other than English is rapidly accelerating, in part due to the influence of large language models and generative artificial intelligence (e.g., GPT estimates of concreteness for Spanish words; Martínez et al., 2025).

Missing data and lexical mismatches (e.g., homophones, polysemes) are additional considerations when using *ConversationAlign*. There are three primary sources of missingness and error variance at the single-word level. First, *ConversationAlign*’s internal lookup database contains static norms for tens of thousands of English words. However, some dimensions have more sparse coverage than others. *ConversationAlign* uses a database join function to tag each content word with its corresponding value in the lookup database. When no published norm exists for a target word, the algorithm returns an NA, denoting a missing value. When a conversation transcript contains many words without corresponding matches in the lookup database, missingness may emerge as a source of bias. A related source of missingness involves procedures for omitting stopwords. *ConversationAlign* (v 0.4.0) contains 4 different stopword lists, each varying in the scope and conservatism of its exclusion criteria. The decision of how to process stopwords is not trivial since many common conversational backchannels (e.g., uh, hm, yeah, okay) are stopwords that, when omitted, will yield NA values, increasing missingness. Although no fixed guidelines exist for missing data, we encourage researchers to report corpus analytics that include missingness along with other descriptive features (e.g., total tokens, type-token ratio, total conversations, average number of turns) as illustrated in Table 1. ConversationAlign includes an automated function that generates many such analytics in a journal-quality table.

An additional source of error variance at the lookup stage involves lexical ambiguity. *ConversationAlign*’s lookup database does not distinguish between homographs, polysemes, or alternate parts-of-speech, instead relying on brute force probability. That is, *ConversationAlign* gambles that the intended meaning of a word in a transcript matches the most frequent variant in English. For example, “bank” more commonly refers to a financial institution rather than the strip of land adjacent to a river. This default “bet” about the intended meaning of a word is agnostic to contextual cues and is a likely source of error variance. Although text disambiguation is improving, its computational demands are prohibitive for *ConversationAlign*, and most databases do not contain separate lexical norms for alternate word forms.

*ConversationAlign* (v 0.4.0) is capable of processing millions of words and computing alignment metrics within just a few minutes. One might, therefore, be tempted to unleash the software to analyze alignment across all possible dimensions (*N* = 45) in a simultaneous model. To our knowledge, there is no universally accepted multiple comparison correction that applies to the nested structure of conversations. *ConversationAlign* (v 0.4.0) does not include a correction option specific to the number of dimensions of interest. However, this does not license a “kitchen sink” approach to analyzing alignment. Users should instead pursue a conservative analysis pipeline that includes pre-registered hypotheses and a small number of theoretically-motivated contrasts. Consider, for example, the case of a language intervention designed to boost morphological complexity in young children. A researcher might test her hypothesis that after treatment, a child will track and successfully align with the morphological complexity of their parent during spontaneous dyads. This is a testable (and falsifiable) hypothesis that would motivate the principled selection of ‘morpheme count’ as the target dimension of interest relative to many other possible dimensions (e.g., concreteness).

The metrics included in *ConversationAlign* (dAUC, correlations) have limitations. Each metric reports only a single value for each conversation (or per offset for correlations), and cannot show changes in language production over time. However, time series data are common, and methods may be implemented with ease. For example, a linear regression on individual interlocutor time series can provide information on how and interlocutor’s production has changed over the course of the conversation. The development of new methods for time series analysis will also provide new applications for the analysis of conversation data with *ConversationAlign*.

## Conclusion

*ConversationAlign* is a novel workflow for modeling linguistic alignment in dyadic conversation transcripts. We demonstrated the use of *ConversationAlign* with a corpus of interviews hosted by Terry Gross as part of the NPR show *Fresh Air*, where we found that Terry Gross and guests showed greater synchrony on ratings of linguistic valence when Terry Gross’s turns were lagged one turn behind relative to leads. This shows that Terry Gross adjusted to her guests’ production more than her guests adjusted to her. This example details how *ConversationAlign* may be used to produce whole-conversation-level metrics of linguistic alignment. However, the pairs of time series produced by *ConversationAlign* on a set of psycholinguistic dimensions also provide a foundation for future, more granular, metrics. *ConversationAlign* is open-source and ready to use upon installation. Future research may apply *ConversationAlign* to different naturalistic conversation settings and contexts. Additionally, *ConversationAlign* may be paired with other methods, including hyperscanning, eye-tracking, and pupillometry. ConversationAlign provides accessible and transparent metrics of alignment, complementing past work and providing new tools for conversation analysis.

## Data Availability

All data are available via the Open Science Foundation at https://osf.io/bkzre/

## Footnotes

1 By our operational definition, lexical alignment (e.g., synchronizing word choices) would be considered one sub-domain of linguistic alignment.

2 This focus on English is not a specific design feature but rather reflects the limited availability of published lexical norms and text-cleaning tools (lemmatization, stopword lists) for languages other than English. We revisit this limitation and the promise of expansion in the general discussion.

3 This list of contractions is bundled into *ConversationAlign* as internal data and can be inspected by visiting the OSF

4 Valence is analogous to pleasantness. Concreteness is the extent to which a word’s referent can be experienced through the senses (e.g., vision, audition, olfaction) (Paivio, 1985). Both variables are typically quantified by subjective ratings.

## Code availability

All code is available via the Open Science Foundation at https://osf.io/bkzre/

## Declarations

### Open practices statement

All data and materials used in analyses reported here are available on OSF (https://osf.io/bkzre/?view_only=). No experiments or analyses were preregistered.
